# Supplementary material for: Multi‐dataset identification of innovative feature genes and molecular mechanisms in keratoconus
Source: J Cell Mol Med. 2024 Sep 19;28(18):e70079. doi: 10.1111/jcmm.70079 (PMC11412914; doi:10.1111/jcmm.70079)
Supplement: Supplementary file 2 — Material S2. [file JCMM-28-e70079-s002.zip › 7.GSEA analysis/6.SNORA74B/KEGG_TOLL_LIKE_RECEPTOR_SIGNALING_PATHWAY.html]

Details for gene set KEGG\_TOLL\_LIKE\_RECEPTOR\_SIGNALING\_PATHWAY[GSEA]

|  || Dataset | SNORA74B.group.cls#h\_versus\_l |
| Phenotype | group.cls#h\_versus\_l |
| Upregulated in class | l |
| GeneSet | KEGG\_TOLL\_LIKE\_RECEPTOR\_SIGNALING\_PATHWAY |
| Enrichment Score (ES) | -0.40791136 |
| Normalized Enrichment Score (NES) | -1.4028672 |
| Nominal p-value | 0.09270217 |
| FDR q-value | 0.33044544 |
| FWER p-Value | 0.895 |
Table: GSEA Results Summary

  

Fig 1: Enrichment plot: KEGG\_TOLL\_LIKE\_RECEPTOR\_SIGNALING\_PATHWAY      
 Profile of the Running ES Score & Positions of GeneSet Members on the Rank Ordered List

  

| SYMBOL | TITLE | RANK IN GENE LIST | RANK METRIC SCORE | RUNNING ES | CORE ENRICHMENT || 1 | PIK3R5 | na | 170 | 0.800 | 0.0409 | No |
| 2 | CD86 | na | 436 | 0.628 | 0.0692 | No |
| 3 | IFNA14 | na | 1314 | 0.437 | 0.0689 | No |
| 4 | IFNA4 | na | 1727 | 0.395 | 0.0797 | No |
| 5 | IL12A | na | 2028 | 0.368 | 0.0921 | No |
| 6 | IFNA6 | na | 2354 | 0.343 | 0.1023 | No |
| 7 | IRF5 | na | 2744 | 0.317 | 0.1093 | No |
| 8 | IFNA16 | na | 3517 | 0.277 | 0.1028 | No |
| 9 | TLR8 | na | 4294 | 0.250 | 0.0947 | No |
| 10 | TRAF3 | na | 7547 | 0.147 | 0.0093 | No |
| 11 | PIK3R1 | na | 8116 | 0.133 | 0.0005 | No |
| 12 | MAPK11 | na | 8296 | 0.129 | 0.0027 | No |
| 13 | IKBKB | na | 8763 | 0.119 | -0.0039 | No |
| 14 | RIPK1 | na | 9049 | 0.112 | -0.0057 | No |
| 15 | CASP8 | na | 9153 | 0.110 | -0.0024 | No |
| 16 | AKT2 | na | 9380 | 0.105 | -0.0029 | No |
| 17 | IRAK1 | na | 10057 | 0.091 | -0.0172 | No |
| 18 | MAPK9 | na | 10427 | 0.085 | -0.0230 | No |
| 19 | TIRAP | na | 10543 | 0.082 | -0.0216 | No |
| 20 | MAPK13 | na | 11114 | 0.073 | -0.0339 | No |
| 21 | LBP | na | 11183 | 0.072 | -0.0317 | No |
| 22 | TOLLIP | na | 11550 | 0.066 | -0.0385 | No |
| 23 | MAPK12 | na | 11855 | 0.062 | -0.0437 | No |
| 24 | TLR5 | na | 12072 | 0.059 | -0.0466 | No |
| 25 | SPP1 | na | 12450 | 0.054 | -0.0544 | No |
| 26 | MAPK14 | na | 12827 | 0.049 | -0.0624 | No |
| 27 | PIK3R2 | na | 12946 | 0.047 | -0.0631 | No |
| 28 | PIK3CA | na | 13079 | 0.046 | -0.0643 | No |
| 29 | FADD | na | 13177 | 0.045 | -0.0645 | No |
| 30 | STAT1 | na | 14481 | 0.030 | -0.1004 | No |
| 31 | IFNB1 | na | 14716 | 0.028 | -0.1056 | No |
| 32 | MAP2K2 | na | 14904 | 0.025 | -0.1095 | No |
| 33 | MAPK10 | na | 14996 | 0.024 | -0.1108 | No |
| 34 | IFNA2 | na | 15155 | 0.023 | -0.1140 | No |
| 35 | PIK3CB | na | 15769 | 0.017 | -0.1307 | No |
| 36 | MAP2K4 | na | 16268 | 0.012 | -0.1444 | No |
| 37 | CD14 | na | 16315 | 0.011 | -0.1451 | No |
| 38 | CHUK | na | 16402 | 0.010 | -0.1470 | No |
| 39 | MYD88 | na | 16730 | 0.007 | -0.1560 | No |
| 40 | MAPK3 | na | 16753 | 0.007 | -0.1563 | No |
| 41 | MAP2K7 | na | 17007 | 0.005 | -0.1633 | No |
| 42 | CTSK | na | 17313 | 0.001 | -0.1720 | No |
| 43 | IRF7 | na | 17455 | 0.000 | -0.1761 | No |
| 44 | AKT1 | na | 18366 | -0.010 | -0.2018 | No |
| 45 | TLR3 | na | 18747 | -0.014 | -0.2119 | No |
| 46 | PIK3CG | na | 18884 | -0.016 | -0.2150 | No |
| 47 | MAP2K3 | na | 18965 | -0.017 | -0.2163 | No |
| 48 | TBK1 | na | 19262 | -0.020 | -0.2237 | No |
| 49 | FOS | na | 19844 | -0.027 | -0.2389 | No |
| 50 | RAC1 | na | 19881 | -0.027 | -0.2384 | No |
| 51 | PIK3CD | na | 20354 | -0.032 | -0.2502 | No |
| 52 | MAPK8 | na | 20414 | -0.033 | -0.2500 | No |
| 53 | TAB1 | na | 20492 | -0.034 | -0.2503 | No |
| 54 | MAPK1 | na | 20606 | -0.035 | -0.2516 | No |
| 55 | IRF3 | na | 20697 | -0.036 | -0.2521 | No |
| 56 | IKBKG | na | 20820 | -0.038 | -0.2534 | No |
| 57 | IFNAR1 | na | 22120 | -0.053 | -0.2879 | No |
| 58 | TAB2 | na | 22232 | -0.054 | -0.2880 | No |
| 59 | IRAK4 | na | 22391 | -0.057 | -0.2893 | No |
| 60 | TRAF6 | na | 22560 | -0.059 | -0.2908 | No |
| 61 | IL12B | na | 22622 | -0.060 | -0.2891 | No |
| 62 | MAP3K7 | na | 23787 | -0.078 | -0.3183 | No |
| 63 | JUN | na | 25317 | -0.104 | -0.3565 | No |
| 64 | CD80 | na | 26418 | -0.126 | -0.3811 | No |
| 65 | NFKB1 | na | 26534 | -0.128 | -0.3770 | No |
| 66 | TLR1 | na | 26542 | -0.129 | -0.3698 | No |
| 67 | IFNAR2 | na | 27194 | -0.144 | -0.3804 | No |
| 68 | CXCL11 | na | 27958 | -0.164 | -0.3931 | No |
| 69 | IFNA7 | na | 28473 | -0.179 | -0.3977 | Yes |
| 70 | NFKBIA | na | 28570 | -0.182 | -0.3901 | Yes |
| 71 | IKBKE | na | 28671 | -0.185 | -0.3824 | Yes |
| 72 | TLR2 | na | 28759 | -0.188 | -0.3741 | Yes |
| 73 | IFNA8 | na | 29291 | -0.203 | -0.3778 | Yes |
| 74 | PIK3R3 | na | 29453 | -0.208 | -0.3705 | Yes |
| 75 | RELA | na | 29651 | -0.214 | -0.3640 | Yes |
| 76 | TICAM1 | na | 29652 | -0.214 | -0.3517 | Yes |
| 77 | MAP3K8 | na | 30218 | -0.234 | -0.3546 | Yes |
| 78 | IL1B | na | 30302 | -0.237 | -0.3434 | Yes |
| 79 | LY96 | na | 30905 | -0.263 | -0.3458 | Yes |
| 80 | MAP2K1 | na | 31129 | -0.275 | -0.3365 | Yes |
| 81 | CXCL8 | na | 31337 | -0.285 | -0.3261 | Yes |
| 82 | TLR4 | na | 31714 | -0.307 | -0.3194 | Yes |
| 83 | TNF | na | 31738 | -0.308 | -0.3024 | Yes |
| 84 | MAP2K6 | na | 31770 | -0.311 | -0.2855 | Yes |
| 85 | IFNA10 | na | 31873 | -0.316 | -0.2703 | Yes |
| 86 | IL6 | na | 32098 | -0.328 | -0.2580 | Yes |
| 87 | CCL5 | na | 32237 | -0.337 | -0.2427 | Yes |
| 88 | CCL3 | na | 32264 | -0.338 | -0.2241 | Yes |
| 89 | CD40 | na | 32420 | -0.349 | -0.2086 | Yes |
| 90 | TLR6 | na | 33895 | -0.511 | -0.2219 | Yes |
| 91 | IFNA21 | na | 33939 | -0.521 | -0.1933 | Yes |
| 92 | TLR7 | na | 33992 | -0.530 | -0.1644 | Yes |
| 93 | TLR9 | na | 34223 | -0.587 | -0.1374 | Yes |
| 94 | CXCL9 | na | 34585 | -0.776 | -0.1034 | Yes |
| 95 | AKT3 | na | 34657 | -0.880 | -0.0551 | Yes |
| 96 | CXCL10 | na | 34695 | -0.997 | 0.0010 | Yes |
Table: GSEA details [plain text format]

  

Fig 2: KEGG\_TOLL\_LIKE\_RECEPTOR\_SIGNALING\_PATHWAY      
 Blue-Pink O' Gram in the Space of the Analyzed GeneSet

  

Fig 3: KEGG\_TOLL\_LIKE\_RECEPTOR\_SIGNALING\_PATHWAY: Random ES distribution      
 Gene set null distribution of ES for **KEGG\_TOLL\_LIKE\_RECEPTOR\_SIGNALING\_PATHWAY**
